# Proteomics-based identification and validation of novel plasma biomarkers phospholipid transfer protein and mannan-binding lectin serine protease-1 in age-related macular degeneration

**DOI:** 10.1038/srep32548

**Published:** 2016-09-08

**Authors:** Hye-Jung Kim, Seong Joon Ahn, Se Joon Woo, Hye Kyoung Hong, Eui Jin Suh, Jeeyun Ahn, Ji Hyun Park, Na-Kyung Ryoo, Ji Eun Lee, Ki Woong Kim, Kyu Hyung Park, Cheolju Lee

**Affiliations:** 1 Center for Theragnosis, Korea Institute of Science and Technology, Seoul, Korea; 2 Department of Ophthalmology, Seoul National University College of Medicine, Seoul National University Bundang Hospital, Seongnam, Korea; 3 Department of Ophthalmology, Hanyang University College of Medicine, Hanyang University Hospital, Seoul, Korea; 4 Department of Ophthalmology, Seoul Metropolitan Government Seoul National University Boramae Medical Center, Seoul, Korea; 5 Department of Neuropsychiatry, Seoul National University Bundang Hospital, Seongnam, Korea; 6 Department of Psychiatry, Seoul National University College of Medicine, Seoul, Korea; 7 Department of Brain and Cognitive Science, Seoul National University College of Natural Sciences, Seoul, Korea

## Abstract

Age-related macular degeneration (AMD) is a major cause of severe, progressive visual loss among the elderly. There are currently no established serological markers for the diagnosis of AMD. In this study, we carried out a large-scale quantitative proteomics analysis to identify plasma proteins that could serve as potential AMD biomarkers. We found that the plasma levels of phospholipid transfer protein (PLTP) and mannan-binding lectin serine protease (MASP)-1 were increased in AMD patients relative to controls. The receiver operating characteristic curve based on data from an independent set of AMD patients and healthy controls had an area under the curve of 0.936 for PLTP and 0.716 for MASP-1, revealing excellent discrimination between the two groups. A proteogenomic combination model that incorporated PLTP and MASP-1 along with two known risk genotypes of *age-related maculopathy susceptibility 2* and *complement factor H* genes further enhanced discriminatory power. Additionally, PLTP and MASP-1 mRNA and protein expression levels were upregulated in retinal pigment epithelial cells upon exposure to oxidative stress *in vitro*. These results indicate that PLTP and MASP-1 can serve as plasma biomarkers for the early diagnosis and treatment of AMD, which is critical for preventing AMD-related blindness.

Age-related macular degeneration (AMD) is clinically characterized by degenerative changes in the macula, the region of the retina specialized for central vision. More than 8 million individuals in the U.S. are estimated to have the disease^1^ and its prevalence is expected to double by the year 2050^2^. A key pathological features of AMD is the development of large drusen, which are tiny yellow or white extracellular deposits located between Bruch’s membrane and the retinal pigment epithelium (RPE). These drusen and associated RPE changes are the major risk factors for the development of advanced AMD, which can be classified into two subtypes—dry (geographic atrophic) and wet/exudative (neovascular)—and can lead to blindness^3^.

Ophthalmic examination or interpretation of fundus photographs by retinal specialists is the main method for diagnosing AMD. Detection of blood biomarkers can be used as an additional screening tool, since blood tests are typically performed at medical check-ups and do not require specialists such as ophthalmologists. Our group recently identified over 320 plasma proteins—including vinculin—that were differentially expressed between AMD patients and healthy controls (HCs) as potential plasma biomarkers^4^.

In this study, we report two candidate biomarkers for AMD, phospholipid transfer protein (PLTP) and mannan-binding lectin (MBL) serine protease (MASP)-1. The diagnostic efficiency of the two proteins was evaluated by enzyme-linked immunosorbent assay (ELISA). In addition, the relationship between plasma PLTP and MASP-1 levels and systemic factors, such as genotype, was analysed. The effect of oxidative stress, which has been linked to AMD, on the expression and/or secretion of PLTP and MASP-1 was also examined.

## Results

### Proteomic analysis of plasma by 4-dimensional (4D) protein profiling

**Figure 1:**
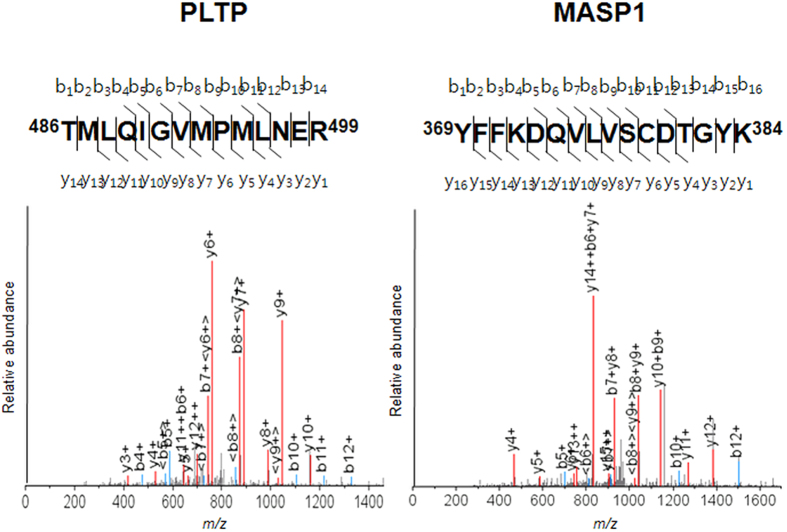
MS/MS profiles of MASP-1 (YFFKDQVLVSCDTGYK) and PLTP (TMLQIGVMPMLNER) peptides; bn and yn denote the fragment ions generated by cleavage of the peptide bond after the *n*th amino acid containing either the N terminus (b series) or C terminus (y series), respectively. The identified peptide sequence location is shown in bold in the protein sequence.

**Figure 2:**
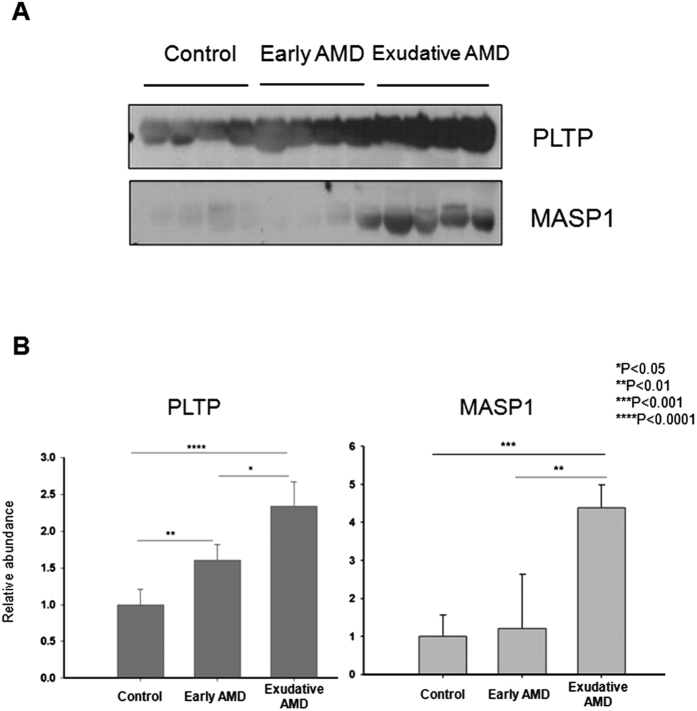
Confirmation of LC-MS/MS data by western blot analysis. (**A**) PLTP and MASP-1 expression in plasma samples from representative randomly selected subjects in healthy control (HC) and early and exudative AMD groups (n = 4, each) for the discovery study. (**B**) Plasma levels of PLTP and MASP-1 were increased in AMD patients relative to HCs in a manner that was dependent on disease severity, as determined by quantitative western blot analysis.

Among the potential biomarkers identified in our large-scale quantitative proteomic analysis, the spectral count of PLTP was >11-fold higher in AMD patients than in HCs, leading us to further investigate this protein in the current study. MASP-1 showed a less dramatic spectral count ratio of 1.13 (AMD:HC), but was also examined here owing to its relationship to signalling pathways implicated in AMD development and progression^5^,^6^,^7^,^8^. Mass spectral characteristics of the two proteins are shown in Fig. 1 and the Supplementary Datasets. A western blot analysis of 12 patients randomly selected from each group revealed an upregulation of PLTP and MASP-1 expression in AMD patients relative to controls that was dependent on disease severity (Fig. 2).

### Plasma concentrations of PLTP and MASP-1 are elevated in AMD

**Figure 3:**
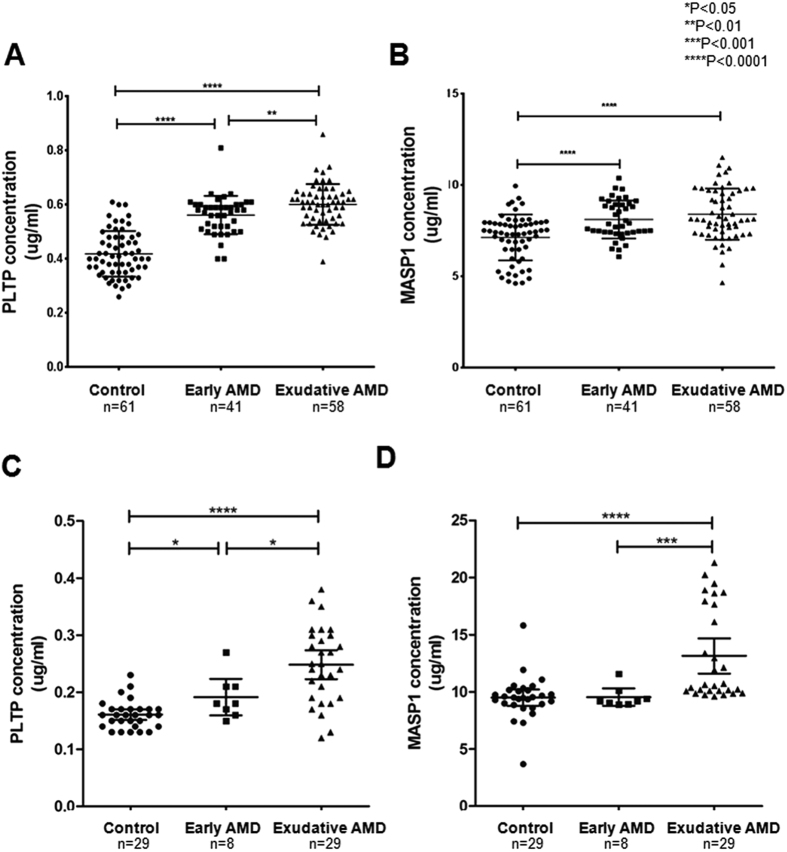
Validation of plasma PLTP and MASP-1 levels in AMD patients and healthy controls (HCs) by ELISA. Concentrations of (**A**) PLTP and (**B**) MASP-1 were measured in samples from HCs (n = 61) and early AMD (n = 41) and exudative AMD (n = 58) patients. (**C**) PLTP and (**D**) MASP-1 levels were validated in a second set of AMD patients and controls (n = 66).

**Figure 4:**
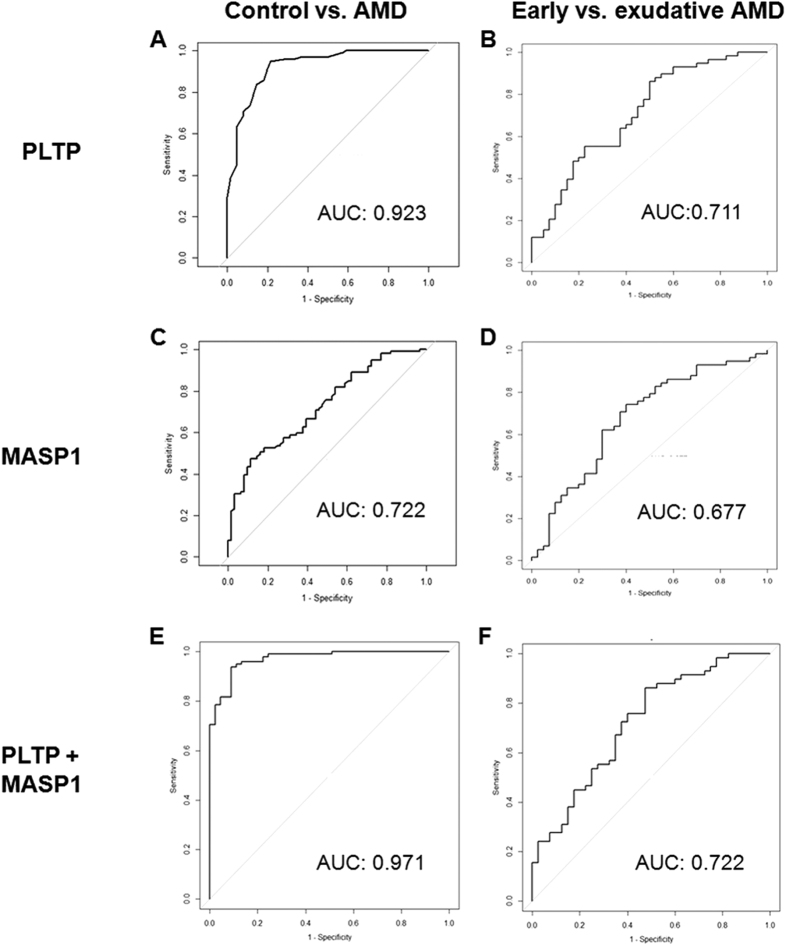
ROC curve of plasma PLTP and MASP-1 levels for detection of early and exudative AMD. AUC values of ROC curves were (**A**) 0.923 for PLTP, (**C**) 0.722 for MASP-1, and (**E**) 0.971 for the two proteins combined between AMD patients and healthy control (HCs). AUC values of ROC curves were (**B**) 0.711 for PLTP, (**D**) 0.677 for MASP-1, and (**F**) 0.722 for the two proteins combined between patients with early and exudative AMD.

Characteristics of patients in the two independent validation sets consisting of HCs and patients with early and exudative AMD are summarized in Table 1. Plasma PLTP and MASP-1 levels in these subjects were evaluated by ELISA (Fig. 3). PLTP and MASP-1 levels were significantly increased in AMD patients relative to controls (P < 0.001 in both validation sets). In addition, PLTP and MASP-1 protein levels were higher in patients with exudative AMD than early AMD. A receiver operating characteristic (ROC) curve based on data from 99 AMD patients and 61 HCs had an area under the curve (AUC) of 0.923 for PLTP and 0.722 for MASP-1 (Fig. 4A,C). The two datasets were combined using a logistic regression model, yielding an AUC of 0.971 for discriminating AMD from HCs (Fig. 4E). As the discriminatory power was mostly attributable to PLTP, these results suggest that this protein can better distinguish between AMD patients and HCs compared to MASP-1. However, neither marker showed good discriminatory power between early and exudative AMD (Fig. 4B,D,F).

The association between clinical variables and plasma PLTP and MASP-1 levels was evaluated by univariate analysis (Table 2). Plasma PLTP level was associated with the presence (P < 0.001) and severity (P = 0.010) of AMD and *age-related maculopathy susceptibility (ARMS*)*2* risk genotype (GT or TT) (P = 0.012), whereas plasma MASP-1 level was associated with the presence of AMD (P < 0.001) and *ARMS2* risk genotype (GT or TT) (P = 0.029). Clinical variables such as age, gender, smoking, diabetes, hypertension, cardiovascular or cerebrovascular incidents, and cancer history were not associated with plasma levels of either protein.

Multivariate regression analysis revealed that plasma levels of both PLTP (regression coefficient = 0.166, P < 0.001) and MASP-1 (regression coefficient = 1.251, P < 0.001) were associated with the presence of AMD. Other clinical variables or genotype data showed no significant association with the plasma levels (Table 3).

### Prediction model combining plasma protein markers and risk genotypes

**Figure 5:**
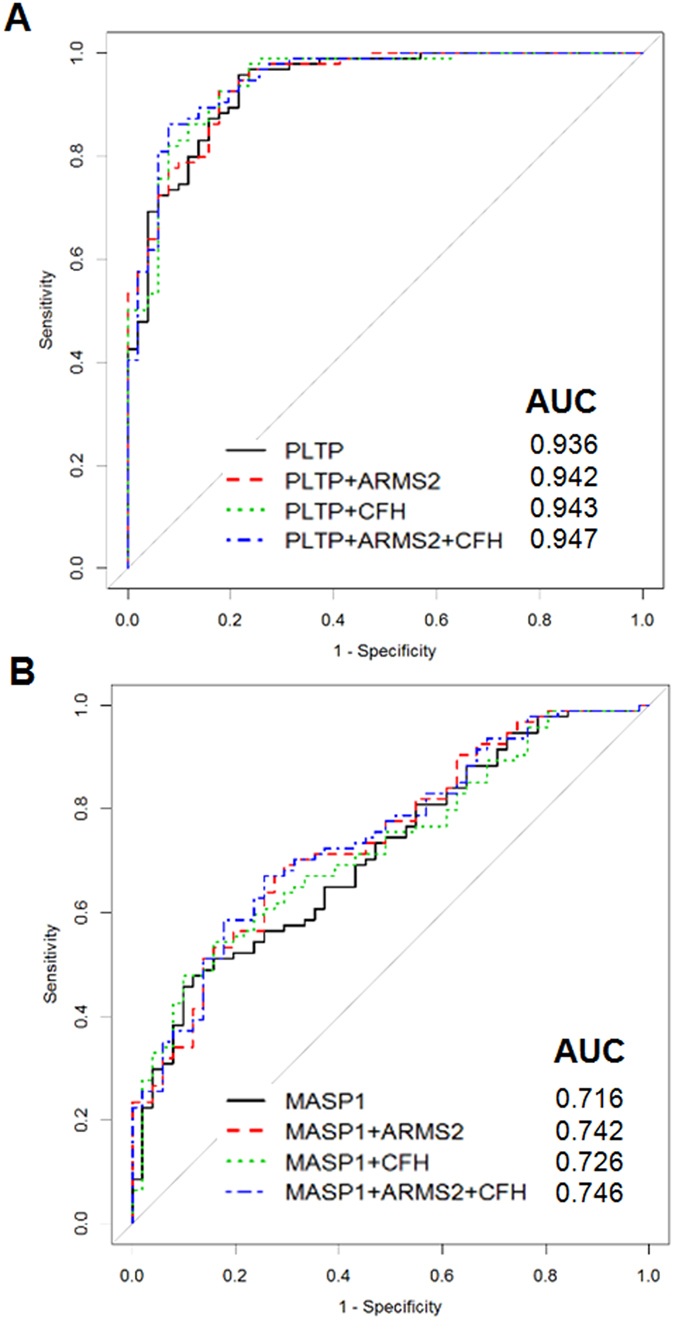
Model combining candidate biomarkers for AMD (PLTP and MASP-1) and two risk SNPs [rs10490924 (ARMS2) and rs800292 (CFH)]. The model showed excellent diagnostic accuracy, especially by PLTP, among 145 AMD patients and healthy control (HCs) for whom genetic information was available.

A proteogenomic prediction model for AMD was generated by combining PLTP and MASP-1 with two known risk single nucleotide polymorphisms (SNPs)—rs10490924 (*ARMS2*) and rs800292 (*CFH*)—that were recently identified as risk alleles for AMD in the Korean population^9^,^10^. The proteogenomic combination model showed slightly better discriminatory power between patients and controls in a validation set of 145 subjects than the model based on proteomic markers only. For example, AUC in the former was 0.947 using PLTP and two SNPs (vs. 0.936 using PLTP only) (Fig. 5A) and 0.746 using MASP-1 and two SNPs (vs. 0.716 using MASP-1 only) (Fig. 5B).

### PLTP and MASP-1 are upregulated in RPE cells exposed to oxidative stress

**Figure 6:**
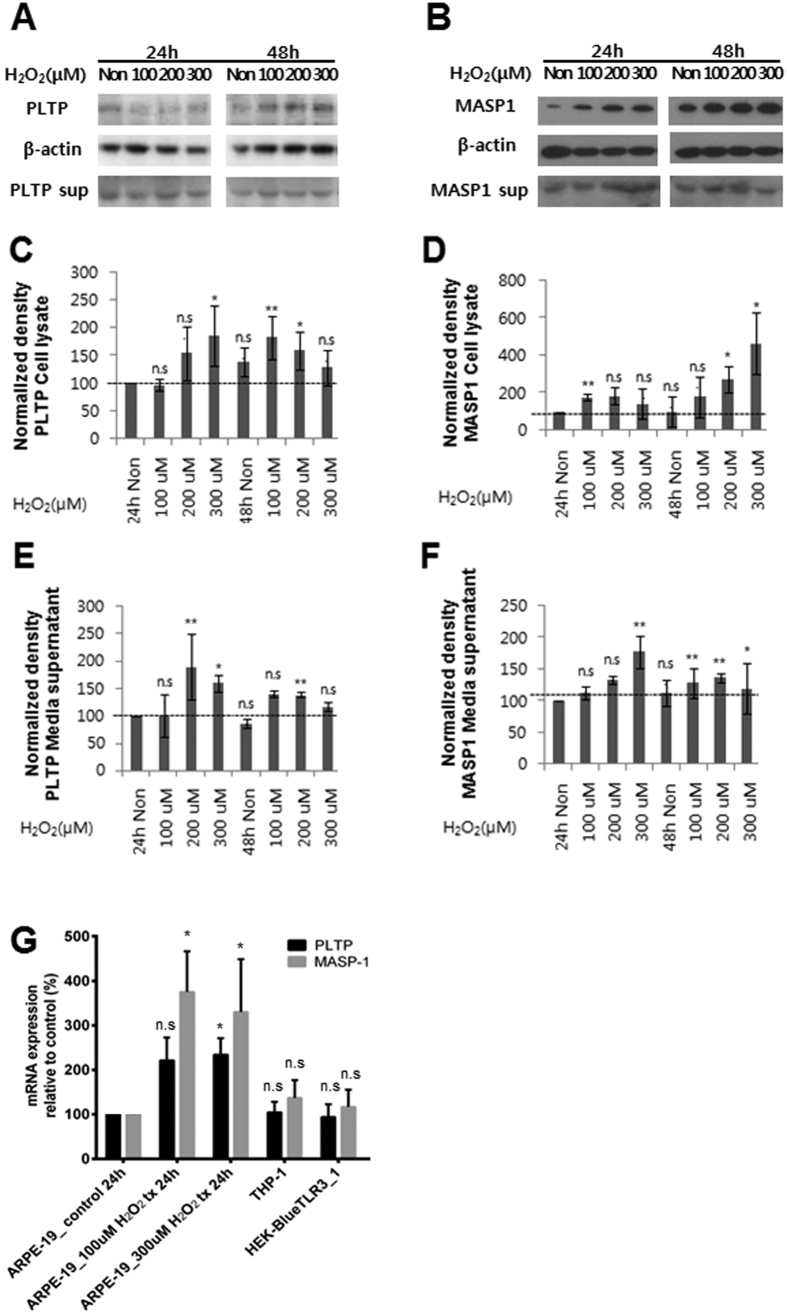
PLTP and MASP-1 expression in ARPE-19 cells exposed to oxidative stress. Western blot analyses of PLTP (**A**,**C**,**E**) and MASP-1 (**B**,**D**,**F**) protein levels. Band intensity was quantified by densitometry, and the paired t test was used to assess differences between groups after intensity values were normalized to the level of β-actin. (**G**) Quantitative RT-PCR analyses of *PLTP* and *MASP-1* mRNA expression in ARPE-19 retinal pigment epithelial cells, THP-1 human monocytes, and human embryonic kidney (HEK) 293 cells under normal growth (control) and oxidative stress (100 or 300 μM H_2_O_2_) conditions.

PLTP and MASP-1 levels were 2- and 4-fold, respectively, higher relative to controls in ARPE-19 cells treated with 300 μm H_2_O_2_, as determined by western blotting (Fig. 6A–D). *PLTP* and *MASP-1* mRNA levels were also elevated upon exposure to oxidative stress in RPE cell lines, as determined by semi-quantitative real-time (RT-)PCR (Fig. 6G).

## Discussion

Proteomic approaches are ideal for identifying novel diagnostic biomarkers^4^,^11^,^12^. Previous proteomic studies of AMD have used ocular tissues such as drusen, RPE/Bruch’s membrane/choroid, and aqueous humour^13^,^14^,^15^,^16^. Despite the potential usefulness of plasma biomarkers for AMD diagnosis, none has been established to date. In the present study, we identified and validated two novel plasma biomarkers for AMD in addition to vinculin from our previous study^4^. This is the first report demonstrating that plasma levels of PLTP and MASP-1 are elevated in AMD patients relative to HCs, providing evidence for the diagnostic capabilities of these biomarkers in AMD detection. At present, diagnosing AMD requires (1) elderly patients visiting a clinic or hospital; (2) fundus examination performed by trained ophthalmologists and retinal specialists; and (3) retinal imaging devices (e.g., fundus angiographic camera and optical coherence tomography). We propose a blood-based diagnostic system for AMD using PLTP and MASP-1 as markers, which would alleviate the above requirements and could potentially be applied anywhere in the world.

In terms of diagnostic accuracy, PLTP showed excellent capacity for discriminating between AMD and control groups (AUC = 0.923) that was superior to that of MASP-1. The diagnostic accuracy was further enhanced in combination with genomic markers or MASP-1, (AUC = 0.947 and 0.971, respectively). However, it is worth noting that neither marker effectively distinguished between early and exudative AMD, which indicate that these novel biomarkers may be useful for AMD screening.

PLTP is an 80-kDa glycoprotein that binds phospholipids and facilitates their transfer between lipoproteins in plasma^17^,^18^ both *in vitro*^19^,^20^,^21^ and *in vivo*^22^. The fact that lipid metabolism is implicated in the pathogenesis of AMD^23^,^24^,^25^ suggests a potential link between this process and PLTP. Recent evidence also indicates that PLTP interacts with proteins linked to immunity and inflammation^26^. Indeed, PLTP levels have been shown to increase during acute inflammation^27^,^28^,^29^, and its activity is associated with the expression of inflammatory markers in patients with type 2 diabetes^30^ and cardiovascular disease^31^, in which systemic inflammation was associated with the disease pathogenesis.

The complement system—a component of the innate immune system—plays an important role in AMD pathogenesis^32^,^33^. MASPs activate the lectin pathway of the complement system upon binding of the pattern recognition molecules MBL and ficolin to target molecules^8^. MASP-2 autoactivates and cleaves complement components (C)2 and 4, generating C3 convertase^34^, which is also known to be generated by MASP-1^6^. The use of monospecific inhibitors confirmed that both MASP-1 and -2 are necessary for lectin pathway activation under physiological conditions^5^,^6^,^7^; indeed, MASP-1 autoactivation is critical for activation of MBL/ficolin complexes^35^. In the present study, we found that the plasma level of MASP-1 was significantly increased in AMD patients’ plasma and the expression of MASP-1 level was increased by oxidative stress in RPE cells, suggesting a previously unreported role for MASP-1 in the pathogenesis of AMD. Furthermore, our findings suggest that the common denominator for PLTP and MASP-1 in terms of function, inflammation, may be an important pathogenic mechanism of AMD.

RPE cells express PLTP and MASP-1, both intra- and extracellularly, implying that these proteins may be secreted into the circulation under oxidative stress conditions in AMD patients. However, it is questionable whether the increase in PLTP and MASP-1 plasma concentration comes from the RPE, as our results cannot draw definite conclusion on the source, and it is also possible that systemic imbalances in lipid metabolism and inflammation lead to elevated plasma concentrations of PLTP and MASP-1 in patients with AMD. Additional studies are necessary to determine the precise origin of these two proteins to explain their increased plasma concentration in patients with AMD.

There were several limitations to our study. Firstly, selection bias and the small number of subjects may have exaggerated the usefulness of PLTP and MASP-1 as diagnostic markers for AMD. Although no selection process was used by investigators, as the study subjects were all consecutive AMD patients or healthy subjects who provided informed consent during the same enrolment period, the patients and controls in our cohort may not be representative of the entire population of AMD patients and healthy controls. Furthermore, the measurement of concentration using commercial ELISA kits has not been fully validated for the novel biomarkers, and may therefore present challenges for medical applications. In particular, we found that PLTP and MASP-1 concentrations varied according to the ELISA kit that was used. Therefore, more accurate and reliable methods for measuring plasma protein concentration are required to validate our findings.

Although noninvasive ophthalmic examination is a more favourable diagnostic method than use of the two plasma biomarkers, there may be specific situations in which the biomarkers could be useful for screening. Examples of these situations include ophthalmologist unavailability or cases where ophthalmic examination including comprehensive fundus examination cannot be performed. As the number of retina specialists is insufficient worldwide, individuals who live in rural areas or underdeveloped countries, in particular, may have limited access to retina specialists, resulting in lower screening rates for AMD. In these situations, screening tests using PLTP and MASP-1 may be useful as the test requires only a small volume of blood. Furthermore, fundus examination to determine the presence of AMD can be unsuitable in cases of media opacity, including cataract. In particular, the elderly population is susceptible to cataract, which significantly impairs fundus examination and subsequently the determination of the presence of AMD. In such cases, although ophthalmic examination is performed, AMD cannot be determined but a blood test might be helpful to indicate the presence of AMD.

In summary, we identified PLTP and MASP-1 as candidate biomarkers for AMD by proteomics analysis and validated the biomarkers using two independent populations of patients and controls. These proteins were linked to inflammation, one of the main pathogenic mechanisms of AMD. Our data showed that both markers, and especially PLTP, had excellent diagnostic accuracy. These along with vinculin can be used for early diagnosis and management of AMD, which is essential for preventing blindness in elderly populations that have limited access to retinal examination services.

## Methods

### Patients and controls

The study was approved by the institutional review board of Seoul National University Bundang Hospital (SNUBH) and followed the tenets of the Declaration of Helsinki. Informed consent was obtained from all subjects. AMD patients were recruited from the retina clinic at the Department of Ophthalmology of SNUBH between January 2008 and January 2014. In the same study period, elderly subjects in the HC group (Age-Related Eye Disease Study [AREDS], Category1^36^) were recruited from the health care centre of SNUBH or from among participants recruited for the population-based longitudinal study described in our previous reports^4^,^37^.

A modification of the AREDS^36^ classification was used by two independent masked reviewers (S.J.A. and J.A.) to grade eyes. We defined no AMD (AREDS Category 1) as no or few small drusen (<63 μm in diameter), early AMD as the presence of one large (≥125 μm in diameter) and many medium-sized (63–124 μm) drusen (AREDS Category 3), and exudative AMD (AREDS Category 4) as neovascular maculopathy characterized by any of the following: choroidal neovascularization, serous and/or haemorrhagic detachment of the neurosensory retina or RPE, retinal hard exudates (a secondary phenomenon resulting from chronic intravascular leakage), subretinal and sub-RPE fibrovascular proliferation, and disciform scarring (subretinal fibrosis). In cases of disagreement between the two reviewers, another senior investigator (S.J.W.) was consulted for the final decision.

In our prior study, individual plasma samples from 20 patients with exudative AMD and 20 age-matched HCs were used for proteome profiling and semi-quantitative comparisons of identified plasma proteins^4^. For validation of novel plasma biomarkers in the current study, 160 samples were collected from 58 patients with exudative AMD, 41 patients with early AMD, and 61 HCs. A total of 66 independent plasma samples from 29 exudative AMD, eight early AMD, and 29 HCs were used for the second validation (Table 1).

### Proteomic analysis of plasma samples by liquid chromatography–tandem mass spectrometry (LC-MS/MS)

Plasma samples were analysed by 4D separation LC-MS/MS as previously described^4^. Detailed information on proteomic experiments, including mass tolerances, software, technical replicates, and statistical analyses, is provided in the Supplementary Methods. Spectral data were searched against the human International Protein Index database (IPI, v.3.44; European Bioinformatics Institute, www.ebi.ac.uk/IPI) using SEQUEST software (TurboSequest v.27, revision 12). Trans-Proteomic Pipeline v.4.0 was used to identify peptides. The total number of spectra was 29,836 for AMD patients and 30,083 for HCs (Supplementary Datasets).

### ELISA

Plasma PLTP and MASP-1 concentrations were measured using commercially available human ELISA kits (MyBioSource, San Diego, CA, USA) according to the manufacturer’s instructions. Absorbance was measured at 450 nm using an ELISA plate reader.

### Western blotting

Plasma samples (10 μg/lane), cell lysate (10 μg/lane), and medium supernatant (30 μl/lane) were size-fractionated by sodium dodecyl sulphate polyacrylamide gel electrophoresis (SDS-PAGE) and transferred to a polyvinylidene difluoride membrane (Amersham Bioscience, Piscataway, NJ, USA), which was blocked with 5% skim milk in Tris-buffered saline with Tween 20 (20 mM Tris, pH 7.4; 150 mM NaCl; 0.05% Tween 20; and 0.01% sodium azide), then incubated at 4 °C overnight with primary antibodies against MASP-1 (Abcam, Cambridge, MA, USA), PLTP, and β-actin (Santa Cruz Biotechnology, Santa Cruz, CA, USA). After washing, the membrane was incubated at 25 °C for 1 h with appropriate horseradish peroxidase-conjugated secondary antibodies, washed, and then developed with chemiluminescence reagent (ECL Plus; GE Healthcare, Uppsala, Sweden). Electroblotting was carried out using a Bio-Rad Trans-Blot Cell system (Bio-Rad, Hercules, CA, USA). Equal loading for each sample was confirmed by Coomassie staining of the remaining portion of SDS-PAGE gels. Protein band intensity was quantified using TotalLab ID v.11.5 (Core Bio, Seoul, Korea). The relative intensities were compared with ImageJ software (National Institutes of Health, Bethesda, MD, USA).

### *In vitro* study using the ARPE-19 cell line

The ARPE-19 human RPE cell line and THP-1 human monocyte cell line were obtained from the American Type Culture Collection (Manassas, VA, USA) and cultured with Dulbecco’s Modified Eagle Medium supplemented with 10% (v/v) heat-inactivated foetal bovine serum, 100 μg/ml streptomycin, and 100 U/ml penicillin at 37 °C in a humidified 5% CO_2_ atmosphere. Cells were treated with 0, 100, 200, 300, 500, or 1000 μM H_2_O_2_ for 24 or 48 h at 37 °C, then washed twice with phosphate-buffered saline and lysed with RIPA Lysis and Extraction Buffer (cat. no. 89900; Thermo Fisher Scientific, Waltham, MA, USA) with protease inhibitors according to the manufacturer’s protocol. Cell lysates were centrifuged at 15,000 × *g* for 10 min at 4 °C and used for western blotting and RT-PCR.

To assess *MASP-1*, *PLTP*, and *glyceraldehyde 3-phosphate dehydrogenase (GAPDH*) mRNA levels, total RNA was isolated from ARPE-19 and THP-1 cells with TRIzol reagent (Invitrogen Life Technologies, Carlsbad, CA, USA). PCR was carried out using the Power SYBR Green RNA-to-CT 1-Step kit, Step One Plus (Applied Biosystems, Foster City, CA, USA) under the following cycling conditions: 48 °C for 30 min, 95 °C for 10 min, 40 cycles of 95 °C for 15 s, and 60 °C for 1 min. The following primers were used: PLTP-F, 5′-TGA CTC CCC ATT GAA GCT GG-3′ and PLTP-R, 5′-CTG GGA CCC GTA CCA TAG TC-3′; MASP-1-F, 5′-AAC ACG GGC TGA TCA CCT TC-3′ and MASP-1-R, 5′-ATC AGC TTC CGG GAG AAC TTG-3′; and GAPDH-F, 5′-ATG GGG AAG GTG AAG GTC G-3′ and GAPDH-R, 5′-GGG GTC ATT GAT GGC AAC AAT A-3′. *MASP-1* and *PLTP* mRNA levels were normalized to that of *GAPDH*.

### Statistical analysis

Plasma concentrations of PLTP and MASP-1 were compared between patients with AMD and healthy controls using Student’s t-test. Univariate regression analyses were performed to evaluate association between clinical variables and plasma PLTP and MASP-1 levels. Statistical significance was defined as P < 0.05. Multivariate regression analyses were performed to identify the factors significantly associated with plasma PLTP and MASP-1 levels. For the analyses, in order to control false positive discovery rate among the multiple clinical and genetic factors, we used Bonferroni correction to set the significance cut-off.

Predictability of novel plasma biomarkers for AMD was evaluated by receiver operating characteristic (ROC) curve analysis. A proteogenomic prediction model for AMD was generated by combining PLTP and MASP-1 with risk SNPs. Among the three SNPs available for our cohort, two SNPs, rs10490924 (*ARMS2*) and rs800292 (*CFH*), were included as they were recently identified as risk alleles for AMD in the Korean population^9^,^10^.

We performed sample size calculation for the second validation set using prior information on PLTP and MASP-1 concentration in the AMD and control groups from the first validation set. The key statistical test was the comparison of PLTP and MASP-1 concentration between AMD patients and controls, and therefore we attempted to include a minimum sample size for each group to detect whether the stated difference existed between the two means. The calculation of sample size was based on the following formula:


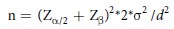


However, to address the possibility of dropouts, unavailable clinical data, or poor quality of fundus photographs leading to difficulties in distinguishing between AMD and controls (potentially up to 20% of assessments), we adjusted sample size to n/(1–0.2). Continuous values are expressed as mean ± standard deviation. All statistical tests were performed using SPSS software (version 18.0; SPSS, Inc., Chicago, IL).

## Additional Information

**How to cite this article**: Kim, H.-J. *et al.* Proteomics-based identification and validation of novel plasma biomarkers phospholipid transfer protein and mannan-binding lectin serine protease-1 in age-related macular degeneration. *Sci. Rep.* **6**, 32548; doi: 10.1038/srep32548 (2016).

## Supplementary Material

Supplementary Table 1

Supplementary Table 2

Supplementary Table 3

Supplementary Information

## Figures and Tables

**Table 1:** Demographics of AMD patients and healthy controls (HCs) used for validation.

Variables	1^st^ validation				2^nd^ validation			
	HC(n = 61)	Early AMD(n = 41)	ExudativeAMD (n = 58)	P value (AMD vs. HC)*	HC(n = 29)	Early AMD(n = 8)	ExudativeAMD (n = 29)	P value (AMD vs. HC)*
Age (years)	75.7 ± 4.7	71.5 ± 6.0	72.2 ± 7.0	**<0**.**001**	77.0 ± 6.4	73.6 ± 7.2	72.1 ± 11.0	0.108
Gender (M:F)	20:41	12:29	23:35	0.740	14:15	0:8	6:23	**<0**.**001**
Systemic risk factors								
Smoking (current or ex-smoker, %)	11† (19.6)	7 (17.1)	19 (32.8)	0.353	11† (40.7)	0	6 (20.7)	0.048
Diabetes (%)	13 (21.3)	15 (36.6)	6 (10.3)	0.988	5† (18.5)	2 (25)	5 (17.2)	0.821
Hypertension (%)	26 (42.6)	21 (51.2)	33 (56.9)	0.143	19† (70.4)	5 (62.5)	15 (51.7)	0.376
Hyperlipidaemia (%)	17 (27.9)	9 (22.0)	17 (29.3)	0.824	2† (33.3)	3 (37.5)	9 (31.0)	1.0
Cardio- or cerebrovascular accident (%)	8 (13.1)	1 (2.4)	2(3.4)	**0**.**022**	1† (3.7)	1 (12.5)	7 (24.1)	0.063
Cancer history (%)	1 (1.6)	3 (7.3)	2 (3.4)	0.409	2 (8.3)	1 (12.5)	0	0.160
Genetic factors (SNP)								
*ARMS2* (rs10490924), G/G:G/T or T/T	24:29	11:30	4:54	**<0**.**001**	N/A	1:7	5:22	1.0
*CFH* (rs1061170), A/A:G/A	46:7	34:7	44:14	0.225	N/A	7:1	18:7	0.643
*CFH* (rs800292), A/A:A/G or G/G	10:43	3:38	10:48	**<0**.**001**	N/A	1:6	2:25	0.511

N/A = not available.

Boldface text indicates statistical significance.

^*^Independent t test, χ^2^ test, or Fisher’s exact test were used where appropriate.

^†^Values were missing for some patients.

**Table 2:** Candidate plasma markers and their association with clinical variables in a validation set of AMD patients and controls.

Clinical variables (number of patients)	PLTP		MASP-1	
	Mean ± standarddeviation (μg/ml)	P value	Mean ± standarddeviation (μg/ml)	P value
Presence of disease, control (61) vs. AMD (99)	0.42 ± 0.08 vs.0.58 ± 0.08	**<0**.**001**	7.14 ± 1.26 vs.8.29 ± 1.27	**<0**.**001**
Disease severity, early (41) vs. exudative AMD (58)	0.56 ± 0.07 vs.0.60 ± 0.08	**0**.**010**	8.12 ± 1.04 vs.8.41 ± 1.41	0.262
Age group, <75 (91) vs. ≥75 years (69)	0.53 ± 0.11 vs.0.51 ± 0.11	0.487	7.84 ± 1.36 vs.7.87 ± 1.41	0.881
Gender, male (55) vs. female (105)	0.53 ± 0.10 vs.0.52 ± 0.12	0.349	7.74 ± 1.29 vs.7.91 ± 1.43	0.464
Smoking, non-smoker (118) vs. current or ex-smoker (29)	0.52 ± 0.12 vs.0.54 ± 0.09	0.257	7.89 ± 1.40 vs.7.85 ± 1.31	0.888
Diabetes mellitus (DM), absent (126) vs. present (34)	0.52 ± 0.11 vs.0.53 ± 0.13	0.826	7.81 ± 1.43 vs.8.02 ± 1.16	0.423
Hypertension (HTN), absent (80) vs. present (80)	0.52 ± 0.10 vs.0.53 ± 0.12	0.549	7.74 ± 1.40 vs.7.97 ± 1.36	0.297
Hyperlipidaemia, absent (117) vs. present (43)	0.52 ± 0.11 vs.0.53 ± 0.13	0.631	7.78 ± 1.32 vs.8.05 ± 1.54	0.316
History of cardio- or cerebrovascular accident, absent (149) vs. present (11)	0.52 ± 0.11 vs.0.48 ± 0.10	0.262*	7.91 ± 1.37 vs.7.10 ± 1.31	0.060*
Cancer history, absent (154) vs. present (6)	0.52 ± 0.11 vs.0.55 ± 0.08	0.507*	7.83 ± 1.38 vs.8.56 ± 1.22	0.205*
*ARMS2* rs10490924, G/G (39) vs. G/T or T/T (113)	0.49 ± 0.11 vs.0.54 ± 0.11	**0**.**012**	7.50 ± 1.31 vs.8.04 ± 1.34	**0**.**029**
*CFH* rs800292, A/A (23) vs. A/G or G/G (129)	0.55 ± 0.09 vs.0.52 ± 0.11	0.375	7.72 ± 1.01 vs.7.94 ± 1.40	0.488

AMD = age-related macular degeneration; ARMS2 = age-related maculopathy susceptibility 2; CFH = complement factor H; MASP-1 = mannan-binding lectin serine protease 1; PLTP = phospholipid transfer protein.

Boldface indicates statistical significance.

^*^Mann-Whitney *U* test.

**Table 3:** Multivariate regression analysis for identification of clinical or SNP data associated with plasma levels of identified biomarkers.

Variables	PLTP		MASP-1	
	Regressioncoefficient	P value	Regressioncoefficient	P value
Age	0.001	0.630	0.037	0.032
Sex	−0.016	0.409	0.183	0.556
AMD vs. control	0.166	**<0**.**001**	1.251	**<0**.**001**
Smoking	−0.009	0.572	−0.072	0.783
*ARMS2* (rs10490924)	−0.010	0.523	0.132	0.607
*CFH* (rs1061170)	0.016	0.344	0.212	0.443
*CFH* (rs800292)	−0.045	0.019	0.078	0.795

AMD = age-related macular degeneration; ARMS2 = age-related maculopathy susceptibility 2; CFH = complement factor H; MASP-1 = mannan-binding lectin serine protease 1; PLTP = phospholipid transfer protein; SNP, single nucleotide polymorphism.

Bonferroni correction was used to set significance level (0.05/14 = 0.0036). Boldface text indicates statistical significance.

## References

[b1] Bressler N. M. *et al.* Potential public health impact of Age-Related Eye Disease Study results: AREDS report no. 11. Archives of ophthalmology 121, 1621–1624, doi: 10.1001/archopht.121.11.1621 (2003). 14609922 PMC1473209

[b2] Rein D. B. *et al.* Forecasting age-related macular degeneration through the year 2050: the potential impact of new treatments. Archives of ophthalmology 127, 533–540, doi: 10.1001/archophthalmol.2009.58 (2009). 19365036

[b3] Ferris F. L. 3rd, Fine S. L. & Hyman L. Age-related macular degeneration and blindness due to neovascular maculopathy. Archives of ophthalmology 102, 1640–1642 (1984). 6208888 10.1001/archopht.1984.01040031330019

[b4] Kim H. J. *et al.* Identification of vinculin as a potential plasma marker for age-related macular degeneration. Investigative ophthalmology & visual science 55, 7166–7176, doi: 10.1167/iovs.14-15168 (2014). 25298412

[b5] Degn S. E. *et al.* Mannan-binding lectin-associated serine protease (MASP)-1 is crucial for lectin pathway activation in human serum, whereas neither MASP-1 nor MASP-3 is required for alternative pathway function. Journal of immunology 189, 3957–3969, doi: 10.4049/jimmunol.1201736 (2012). 22966085

[b6] Heja D. *et al.* Monospecific inhibitors show that both mannan-binding lectin-associated serine protease-1 (MASP-1) and -2 Are essential for lectin pathway activation and reveal structural plasticity of MASP-2. The Journal of biological chemistry 287, 20290–20300, doi: 10.1074/jbc.M112.354332 (2012). 22511776 PMC3370211

[b7] Heja D. *et al.* Revised mechanism of complement lectin-pathway activation revealing the role of serine protease MASP-1 as the exclusive activator of MASP-2. Proceedings of the National Academy of Sciences of the United States of America 109, 10498–10503, doi: 10.1073/pnas.1202588109 (2012). 22691502 PMC3387078

[b8] Thiel S. Complement activating soluble pattern recognition molecules with collagen-like regions, mannan-binding lectin, ficolins and associated proteins. Molecular immunology 44, 3875–3888, doi: 10.1016/j.molimm.2007.06.005 (2007). 17768106

[b9] Kim Y. H., Kim H. S., Mok J. W. & Joo C. K. Gene-gene interactions of CFH and LOC387715/ARMS2 with Korean exudative age-related macular degeneration patients. Ophthalmic genetics 34, 151–159, doi: 10.3109/13816810.2012.749287 (2013). 23289807

[b10] Woo S. J. *et al.* Analysis of Genetic and Environmental Risk Factors and Their Interactions in Korean Patients with Age-Related Macular Degeneration. PloS One 10, e0132771, doi: 10.1371/journal.pone.0132771 (2015). 26171855 PMC4501798

[b11] Hanash S. M., Pitteri S. J. & Faca V. M. Mining the plasma proteome for cancer biomarkers. Nature 452, 571–579, doi: 10.1038/nature06916 (2008). 18385731

[b12] Doecke J. D. *et al.* Blood-based protein biomarkers for diagnosis of Alzheimer disease. Archives of neurology 69, 1318–1325, doi: 10.1001/archneurol.2012.1282 (2012). 22801742 PMC6287606

[b13] Chen H., Liu B., Lukas T. J. & Neufeld A. H. The aged retinal pigment epithelium/choroid: a potential substratum for the pathogenesis of age-related macular degeneration. PloS one 3, e2339, doi: 10.1371/journal.pone.0002339 (2008). 18523633 PMC2394659

[b14] Crabb J. W. *et al.* Drusen proteome analysis: an approach to the etiology of age-related macular degeneration. Proceedings of the National Academy of Sciences of the United States of America 99, 14682–14687, doi: 10.1073/pnas.222551899 (2002). 12391305 PMC137479

[b15] Kim T. W. *et al.* Proteomic analysis of the aqueous humor in age-related macular degeneration (AMD) patients. Journal of proteome research 11, 4034–4043, doi: 10.1021/pr300080s (2012). 22702841

[b16] Nordgaard C. L. *et al.* Proteomics of the retinal pigment epithelium reveals altered protein expression at progressive stages of age-related macular degeneration. Investigative ophthalmology & visual science 47, 815–822, doi: 10.1167/iovs.05-0976 (2006). 16505012

[b17] Albers J. J. *et al.* Functional expression of human and mouse plasma phospholipid transfer protein: effect of recombinant and plasma PLTP on HDL subspecies. Biochimica et biophysica acta 1258, 27–34 (1995). 7654777 10.1016/0005-2760(95)00091-p

[b18] Desrumaux C. M. *et al.* Phospholipid transfer protein is present in human atherosclerotic lesions and is expressed by macrophages and foam cells. Journal of lipid research 44, 1453–1461, doi: 10.1194/jlr.M200281-JLR200 (2003). 12730304

[b19] Albers J. J. & Cheung M. C. Emerging roles for phospholipid transfer protein in lipid and lipoprotein metabolism. Current opinion in lipidology 15, 255–260 (2004). 15166780 10.1097/00041433-200406000-00004

[b20] Tall A. R., Krumholz S., Olivecrona T. & Deckelbaum R. J. Plasma phospholipid transfer protein enhances transfer and exchange of phospholipids between very low density lipoproteins and high density lipoproteins during lipolysis. Journal of lipid research 26, 842–851 (1985). 4031662

[b21] Tollefson J. H., Ravnik S. & Albers J. J. Isolation and characterization of a phospholipid transfer protein (LTP-II) from human plasma. Journal of lipid research 29, 1593–1602 (1988). 2854151

[b22] Van Tol A., Jauhiainen M., De Crom R. & Ehnholm C. Role of phospholipid transfer protein in high-density lipoprotein metabolism: insights from studies in transgenic mice. International journal of tissue reactions 22, 79–84 (2000). 10937357

[b23] Curcio C. A., Johnson M., Huang J. D. & Rudolf M. Apolipoprotein B-containing lipoproteins in retinal aging and age-related macular degeneration. Journal of lipid research 51, 451–467, doi: 10.1194/jlr.R002238 (2010). 19797256 PMC2817575

[b24] Cougnard-Gregoire A. *et al.* Elevated high-density lipoprotein cholesterol and age-related macular degeneration: the Alienor study. PloS one 9, e90973, doi: 10.1371/journal.pone.0090973 (2014). 24608419 PMC3946623

[b25] Ebrahimi K. B. & Handa J. T. Lipids, lipoproteins, and age-related macular degeneration. Journal of lipids 2011, 802059, doi: 10.1155/2011/802059 (2011). 21822496 PMC3147126

[b26] Cheung M. C., Vaisar T., Han X., Heinecke J. W. & Albers J. J. Phospholipid transfer protein in human plasma associates with proteins linked to immunity and inflammation. Biochemistry 49, 7314–7322, doi: 10.1021/bi100359f (2010). 20666409 PMC2930196

[b27] Barlage S. *et al.* ApoE-containing high density lipoproteins and phospholipid transfer protein activity increase in patients with a systemic inflammatory response. Journal of lipid research 42, 281–290 (2001). 11181759

[b28] Levels J. H. *et al.* Alterations in lipoprotein homeostasis during human experimental endotoxemia and clinical sepsis. Biochimica et biophysica acta 1771, 1429–1438, doi: 10.1016/j.bbalip.2007.10.001 (2007). 17980169

[b29] Pussinen P. J. *et al.* The role of plasma phospholipid transfer protein (PLTP) in HDL remodeling in acute-phase patients. Biochimica et biophysica acta 1533, 153–163 (2001). 11566452 10.1016/s1388-1981(01)00153-6

[b30] Rai A. J. *et al.* HUPO Plasma Proteome Project specimen collection and handling: towards the standardization of parameters for plasma proteome samples. Proteomics 5, 3262–3277, doi: 10.1002/pmic.200401245 (2005). 16052621

[b31] Cheung M. C. *et al.* Phospholipid transfer protein activity is associated with inflammatory markers in patients with cardiovascular disease. Biochimica et biophysica acta 1762, 131–137, doi: 10.1016/j.bbadis.2005.09.002 (2006). 16216472

[b32] Nussenblatt R. B., Liu B. & Li Z. Age-related macular degeneration: an immunologically driven disease. Curr Opin Investig Drugs 10, 434–442 (2009). 19431076

[b33] Patel M. & Chan C. C. Immunopathological aspects of age-related macular degeneration. Seminars in immunopathology 30, 97–110, doi: 10.1007/s00281-008-0112-9 (2008). 18299834 PMC2441602

[b34] Gal P. *et al.* A true autoactivating enzyme. Structural insight into mannose-binding lectin-associated serine protease-2 activations. The Journal of biological chemistry 280, 33435–33444, doi: 10.1074/jbc.M506051200 (2005). 16040602

[b35] Megyeri M. *et al.* Quantitative characterization of the activation steps of mannan-binding lectin (MBL)-associated serine proteases (MASPs) points to the central role of MASP-1 in the initiation of the complement lectin pathway. The Journal of biological chemistry 288, 8922–8934, doi: 10.1074/jbc.M112.446500 (2013). 23386610 PMC3610966

[b36] Age-Related Eye Disease Study Research. G. A randomized, placebo-controlled, clinical trial of high-dose supplementation with vitamins C and E, beta carotene, and zinc for age-related macular degeneration and vision loss: AREDS report no. 8. Archives of ophthalmology 119, 1417–1436 (2001). 11594942 10.1001/archopht.119.10.1417 PMC1462955

[b37] Jhoo J. H. *et al.* Prevalence of dementia and its subtypes in an elderly urban korean population: results from the Korean Longitudinal Study on Health And Aging (KLoSHA). Dementia and geriatric cognitive disorders 26, 270–276, doi: 10.1159/000160960 (2008). 18841012

