# Supplementary material for: Proteomics-based identification and validation of novel plasma biomarkers phospholipid transfer protein and mannan-binding lectin serine protease-1 in age-related macular degeneration
Source: Sci Rep. 2016 Sep 8;6:32548. doi: 10.1038/srep32548 (PMC5015054; doi:10.1038/srep32548)

Supplementary Information

Proteomics-based identification and validation of novel plasma biomarkers phospholipid transfer protein and mannan-binding lectin serine protease-1 in age-related macular degeneration

Hye-Jung Kim, Seong Joon Ahn, Se Joon Woo, Hye Kyoung Hong, Eui Jin Suh, Jeeyun Ahn, Ji Hyun Park, Na-Kyung Ryoo, Ji Eun Lee, Ki Woong Kim, Kyu Hyung Park, and Cheolju Lee

Supplementary methods

4D separation liquid chromatography tandem mass spectrometry

Immunoaffinity depletion of major plasma proteins from patients and controls

The top six most abundant proteins (serum albumin, immunoglobulin G, immunoglobulin A, transferrin, haptoglobin, and antitrypsin) were depleted using a MARS column (Agilent Technology, Santa Clara, CA, USA)[1]. Plasma (40 µL) was diluted 1:5 in a proprietary "buffer A" and loaded onto the MARS column. The unbound fraction was concentrated by ultrafiltration using a Microcon filter (3000-Da cutoff; Millipore, MA, USA).

Gel-eluted liquid fraction entrapment electrophoresis (GELFrEE)

Protein concentrations were determined by the Bradford method. Subsequently, an equal amount of concentrated unbound fraction from each healthy control (HC) sample and equal amounts of protein from each age-related macular degeneration (AMD) sample were mixed separately. Further details on the GELFrEE system can be obtained from publications by Tran et al[2,3]. A total of 500 µg of protein was loaded onto a 1 cm gel column, cast to 12% T (with a 3-cm stacking gel cast to 4% T) and electrophoresed at 240 V for 2 h. Following elution of the dye front (fraction 'zero'), additional collections were performed at 5, 15, 30, 50, and 120 min, yielding a total of five fractions.

Isoelectrofocusing of tryptic peptides

Ten GELFrEE fractions (five from HC and five from AMD) were adjusted to the same protein concentration, reduced with 10 mM DTT for 30 min at 37°C, and alkylated with 40 mM iodoacetamide for 1 h in the dark at 25°C. After the samples were diluted 10-fold with 50 mM NH_4HCO_3, trypsin was added at a ratio of 1:40 (w:w), followed by incubation overnight at 37°C. A 3100 OFFGEL Fractionator and OFFGEL Kit pH 3–10 (Agilent Technologies) in a 12-well format were used for isoelectrofocusing of the tryptic peptides.

Peptides were diluted in 1.8 mL of focusing buffer containing only 5% (v/v) glycerol, which deviated from the supplier's protocol. Immobilized pH gradient (IPG) strips were rehydrated by adding 40 μL of peptide IPG strip rehydration solution per well for 15 min. Next, 150 μL of sample was loaded into each well. Peptide focusing was performed until a voltage of 20 kVh was reached, with maximum voltage of 8000 V and maximum current of 50 μA. After focusing, 12 peptide fractions and both ends (total: 14 fractions) were withdrawn, and wells were rinsed with 150 μL of H_2O/MeOH/TFA (49/50/1 v/v) for 15 min. Rinsing solutions were pooled with their corresponding peptide fractions and concentrated in a SpeedVac prior to liquid chromatography tandem mass spectrometry (LC-MS/MS) analysis.

Reversed phase LC-MS/MS analysis

LC-MS/MS was performed on an Agilent nanoflow-1200 series HPLC system connected to a linear ion trap mass spectrometer (LTQ, Thermo Scientific, San Jose, CA, USA). Peptides were reconstituted with 10 μL of 0.4% acetic acid, after which an aliquot (1 μL) was injected into a reversed phase Magic C18aq column (12 cm × 75 μm) equilibrated with 95% solvent A (0.1% formic acid in H_2O) + 5% solvent B (0.1% formic acid in acetonitrile). The peptides were eluted in a linear gradient of 10% to 30% solvent B over 80 min. The MS survey was scanned from 300 to 2000 *m/z*, followed by six data-dependent MS/MS scans with the following options: isolation width, 1.5 *m/z*; normalized collision energy, 25%; and dynamic exclusion duration, 180 s. In total, 140 LC-MS/MS runs (2 pooled samples × 5 GELFrEE fractions × 14 OFFGEL fractions × single technical replicate) were performed. The acquired MS/MS spectra were compared against the human International Protein Index database, including known contaminants (IPI, version 3.44, European Bioinformatics Institute, www.ebi.ac.uk/IPI), using SEQUEST (TurboSequest version 27, revision 12) with a mass tolerance of ± 0.5 Da for MS/MS and ± 3.0 Da for MS. Searches were performed with the

options of no enzyme, a fixed modification of carbamidomethylation at cysteine (+57 Da), and a variable modification of methionine oxidation (++16 Da). Peptide assignment was performed with the Trans-Proteomic Pipeline (TPP, version 4.0, http://www.proteomecenter.org). The SEQUEST search output was used as the input for Peptide-Prophet and Protein-Prophet. Peptides with probabilities greater than 0.05 were included in the subsequent Protein-Prophet analysis, and proteins having protein probabilities of more than 0.9 were gathered. This probability corresponded to an FDR of 0.007 for the healthy control dataset and an FDR of 0.012 for the AMD dataset.

Quantification and statistical analysis

The relative abundance of identified proteins was estimated on the basis of spectral counts (the number of MS/MS spectra matched to the protein of interest). Spectral count data were extracted using the Protein Prophet program and exported to MS-EXCEL. Afterward, the normalized spectral abundance factor (NSAF) for each protein was obtained considering the total numbers of spectra for AMD patients and HCs (29,836 and 30,083, respectively)[4,5]. We calculated an NSAF for each protein as follows:

$$(\mathrm{NSAF})_k = \frac{(\mathrm{SpC}/L)_k}{\sum_{i=1}^{N} (\mathrm{SpC}/L)_i}$$

in which the total number of tandem MS spectra-matching peptides from protein k (SpC) was divided by the protein length (L) and then divided by the sum of SpC/L for all N proteins. The abundance ratio for each protein was calculated as protein ratio = (average NSAF of the protein in AMD) / (average NSAF of the protein in HC).

In order to filter out differentially expressed proteins (DEPs) that were statistically significant, G-tests adjusted by the William's correction were performed. The spectral counts for each protein were first normalized as

$$n(\mathrm{AMD})_{\mathrm{i}} = (\mathrm{SpC})_{i\,\in \mathrm{AMD}} \frac{\sum_{j\,\in \mathrm{AMD}} (\mathrm{SpC})_j}{\sum_{k\,\in \mathrm{AMD}} (\mathrm{SpC})_k} + 0.5$$

$$n(\mathrm{HC})_{\mathrm{i}} = (\mathrm{SpC})_{i\,\in \mathrm{HC}} + 0.5$$

where $(\mathrm{SpC})_{i\in \mathrm{AMD}}$ and $(\mathrm{SpC})_{i\in \mathrm{HC}}$ denote spectral counts of the i-th protein from the AMD and HC groups, respectively, and $\sum_{j\in \mathrm{AMD}}(\mathrm{SpC})_j$ and $\sum_{k\in \mathrm{AMD}}(\mathrm{SpC})_k$ are the total numbers of spectra. The G-value for the i-th protein was then calculated as follows:

$$G_i = 2\ln\left(\frac{2n(\mathrm{AMD})_i}{n(\mathrm{AMD})_i + n(\mathrm{HC.})_i}\right) n(\mathrm{AMD})_i + 2\ln\left(\frac{2n(\mathrm{HC})_i}{n(\mathrm{AMD})_i + n(\mathrm{HC})_i}\right) n(\mathrm{HC})_i$$

A G-value higher than 3.841 was considered significant, with $P < 0.05$ according to the χ^2-distribution[7]. In addition, the rank-sum test (Mann-Whitney test) was used to evaluated the difference in protein expression between AMD patients and HCs. All statistical analyses were performed using MedCalc (version 9.6.2.0, MedCalc Software, Ostend, Belgium).

REFERENCES

1. **Kim HJ, *et al.* (2009) Identification of S100A8 and S100A9 as serological markers for colorectal cancer. *J Proteome Res* 8(3):1368-1379.**
2. **Lee JE, *et al.* (2009) A robust two-dimensional separation for top-down tandem mass spectrometry of the low-mass proteome. *J Am Soc Mass Spectrom* 20(12):2183-2191.**
3. **Tran JC & Doucette AA (2008) Gel-eluted liquid fraction entrapment electrophoresis: an electrophoretic method for broad molecular weight range proteome separation. *Anal Chem* 80(5):1568-1573.**
4. **Paoletti AC, *et al.* (2006) Quantitative proteomic analysis of distinct mammalian Mediator complexes using normalized spectral abundance factors. *Proc Natl Acad Sci U S A* 103(50):18928-18933.**
5. **Yang WS, *et al.* (2012) Proteomic approach reveals FKBP4 and S100A9 as potential prediction markers of therapeutic response to neoadjuvant chemotherapy in patients with breast cancer. *J Proteome Res* 11(2):1078-1088.**
6. **Zhang B, *et al.* (2006) Detecting differential and correlated protein expression in label-free shotgun proteomics. *J Proteome Res* 5(11):2909-2918.**

Supplementary Figure S1. A receiver operating characteristic (ROC) curve of plasma PLTP and MASP-1 levels for detection of age-related macular degeneration (AMD) in the second validation set

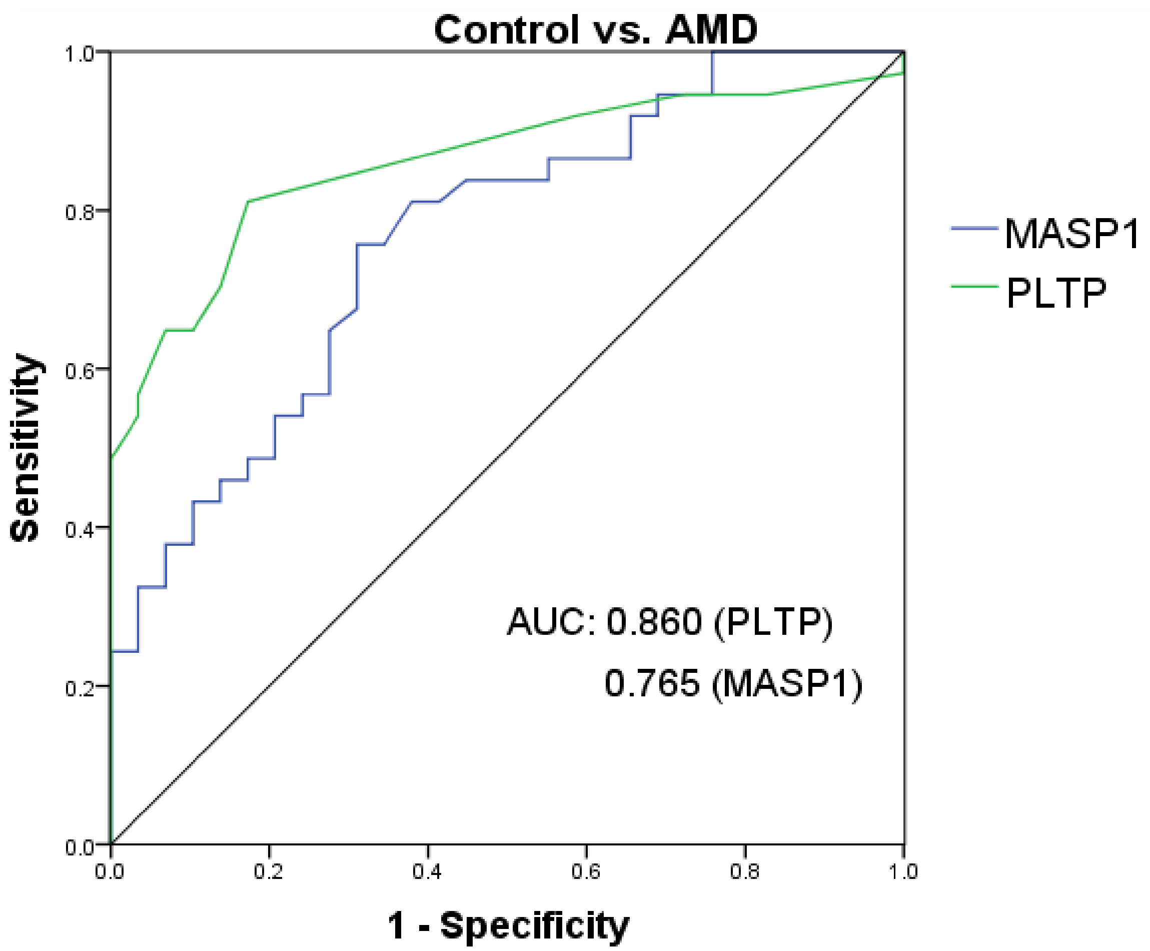

Supplementary Figure S2. A volcano plot demonstrating magnitude and significance of the protein comparisons between groups. The vertical axis indicates -log10(p-value). The horizontal axis indicates log2 fold change.

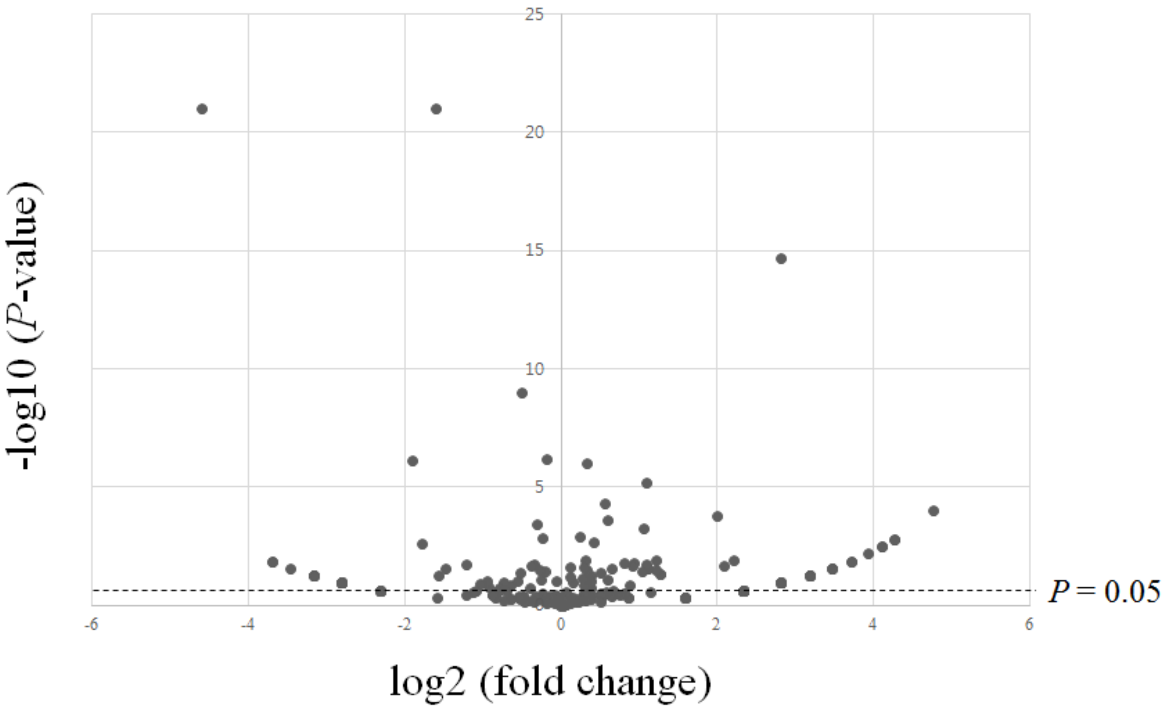

Supplement: Supplementary Information [file srep32548-s4.pdf]
